# Removal of huge hook from a boy's scrotum without any surgical intervention: A case report

**DOI:** 10.1016/j.eucr.2021.101857

**Published:** 2021-09-16

**Authors:** Serajoddin Vahidi, Hojat Alipoor, Mohsen Ahrari, Saeid Abouei

**Affiliations:** a Department of Urology, Shahid Sadoughi University of YAZD, Iran; b Resident of Urology,department of Urology, Shahid Sadoughi University of Medical Sciences, Iran; c Student Research Committee, Faculty of Medicine, Shahid Sadoughi University of Medical Sciences, Yazd, Iran

**Keywords:** Forign body, Genitalia, Scrotum, Trauma

## Abstract

A significant proportion of abdominal and pelvic injuries in men are the genitourinary system, which involves the external genitalia more than the others. Due to the specific anatomical position of the external genitourinary and the participation of men in activities such as martial arts, violent interaction and war activities, the incidence of injury is higher than women. In this case report, we report the traumatic incident of an 11-year-old boy whose boxing bag fell while moving, hooked his perineum and exit from the suprapubic.

## 1 Introduction

The significant proportion of abdominal and pelvic injuries in men are due to their genitourinary system, which a large proportion of these events are relate to the external genitalia. Due to the special anatomical position of the male reproductive system (scrotum and testicles) the genitourinary system injuries are more common in men. The most important point is how to deal with these patients.^1^ These injuries, as well as their consequences, can affect men at any age.^2^ Incidents such as urethra and testicle rupture, as well as internal scrotum and testicular bleeding are the consequences of testicle trauma.^3^ In some cases, penetrating trauma such as knife, bullet, or a sharp-edged object can damage the scrotum, urethra, and testicles. A general principle for assessing penetrating scrotal trauma, especially with severe injury, is early detection and repair operation.^1^ Faster pain relief, control of bleeding, examination of testicular and ductal damage, and prevention of secondary consequences are the benefits of primary exploration.

In this report, we present a case of penetrating scrotum trauma that has been managed with minimal surgery and had excellent results.

## 2 Case report

**Fig. 1:**
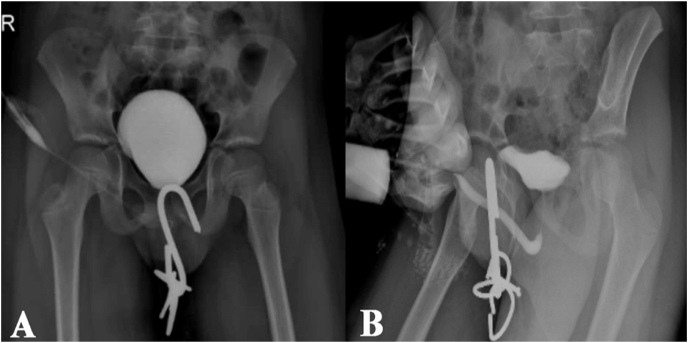
A) Cystography of the patient B) RUG of the patient.

**Fig. 2:**
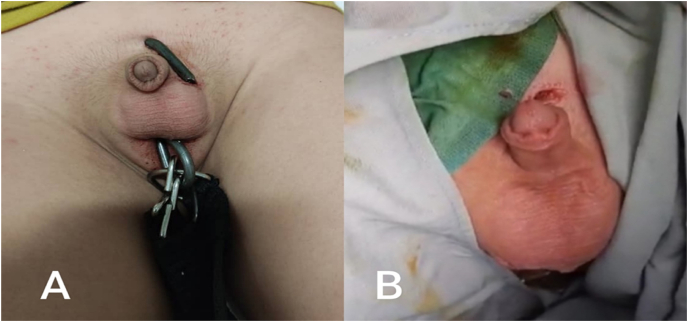
Picture of scrotum-penetrating trauma A) Before extracting the foreign body B) After extracting the foreign body.

An 11-year-old boy was moving his boxing bag, it accidently fell off and causing the large hook entering the bottom of scrotum and exit from suprapubic. The boy was taken to the emergency ward by his anxious parents, suffering from severe pain. No active bleeding, irritation or obstructive urinary symptoms were observed. Emergency ultrasound was performed showing no testicular rupture and scrotum hematoma, followed by RUG and cystography in different positions to rule out urethral and bladder damage (Fig. 1). The patient was then taken to the operating room for removing the external object from the scrotum. After general anesthesia he underwent a lithotomy position and Urethrocystoscopy was performed by rigid cystoscope (8fr). Although, the external object was moved longitudinally from the lower part of scrotum to the suprapubic section, fortunately there was no evidence of urethra or bladder damage due to lack of urethra and scrotum. The external object was then easily removed from inferior testis with manipulation in the direction of its own structure without any surgical incision. The patient was able to spontaneous urination without any blood on meatus (Fig. 2). For disinfection, the wound was washed by diluted betadine and normal saline; the edge of wound was scratched which was negligibleand not sutured to induce discharge possibility. The boy was then transferred to the ward due to stable vital signs with testicular elevation and wound bandage. The patient was hospitalized for six days and being treated with ceftriaxone and clindamycin. UA, UC, and CBC were prescribed and all proved normal. The patient was discharged from the hospital, observing no discharge from the local wound and scrotum hematoma, and with administration of Cefixine and daily washing. By one-week follow-up, the patient had normal evaluation without any evidences of hematoma, infection or complication.

## 3 Discussion

To evaluate the injury inside and outside the scrotum and to simultaneously assess the type of trauma the history and physical examination is important. Other paraclinical findings, such as laboratory tests (e.g., CBC/UA), may also help managing the case, especially in the resuscitation phase.^1^ Unfortunately, the physical examination in the acute post-traumatic scrotum can be difficult and unreliable for a variety of reasons (e.g., non-co-operative patient due to significant pain and soft tissue swelling).^3^ Lack of reliable physical examination may lead to early operative exploration. In the first stage, scrotum ultrasound can explicitly assess the scrotum damage and diagnose the testicular rupture with high accuracy. With Doppler flow technique, the imaging method for external urogenital lesions is high-frequency ultrasound. Ultrasound is used to assess the integrity of damaged tissue in testicular trauma and is readily available and inexpensive. For these reasons, ultrasound is useful in following up patients after scrotal trauma.^4^ An important point in the penetrating trauma management (especially when the urethra and testicles are not damaged) is the negligence and lack of attention to the high risk of infection and necrosis at the site of trauma, which requires careful follow-up and re-imaging.^1^ MRI is another reliable method of imaging in patients with penetrating scrotal trauma. In penetrating scrotum trauma, the rate of testicular non-damage is significantly lower (ranging from 32 to 65%),^5^ as fortunately was observed in our case; the testicles and urethra were not damaged; the ultrasound and imaging findings fully confirmed this. The patient was then treated with great care but did not inclined to be candidate for early operative exploration. However, to remove the external object, and to confirm the clinical findings and imaging, the patient was transferred to the operating room and ureterocystoscopy was performed which is normal. The important point is not suturing in the inlet and outlet of the external object helped to prevent the infection. In conclusion, urologists can use imaging techniques in scrotal traumas due to the ease and safety of the procedure to determine possible injuries for definitive diagnosis, and consider, the appropriateness of the external object entry size to prevent suture and infection eventually.
